# The Glasgow Norms: Ratings of 5,500 words on nine scales

**DOI:** 10.3758/s13428-018-1099-3

**Published:** 2018-09-11

**Authors:** Graham G. Scott, Anne Keitel, Marc Becirspahic, Bo Yao, Sara C. Sereno

**Affiliations:** 1 School of Media, Culture and Society–Psychology, University of the West of Scotland, Paisley, PA1 2BE UK; 2 Institute of Neuroscience and Psychology, University of Glasgow, 62 Hillhead Street, Glasgow, G12 8QB UK; 3 School of Psychology, University of Glasgow, 62 Hillhead Street, Glasgow, G12 8QB UK; 4 Division of Neuroscience and Experimental Psychology, University of Manchester, Manchester, UK

**Keywords:** Psycholinguistic norms, Arousal, Valence, Dominance, Concreteness, Imageability, Familiarity, Age of acquisition, Semantic size, Gender association

## Abstract

The Glasgow Norms are a set of normative ratings for 5,553 English words on nine psycholinguistic dimensions: arousal, valence, dominance, concreteness, imageability, familiarity, age of acquisition, semantic size, and gender association. The Glasgow Norms are unique in several respects. First, the corpus itself is relatively large, while simultaneously providing norms across a substantial number of lexical dimensions. Second, for any given subset of words, the same participants provided ratings across all nine dimensions (33 participants/word, on average). Third, two novel dimensions—semantic size and gender association—are included. Finally, the corpus contains a set of 379 ambiguous words that are presented either alone (e.g., *toast*) or with information that selects an alternative sense (e.g., *toast (bread)*, *toast (speech)*). The relationships between the dimensions of the Glasgow Norms were initially investigated by assessing their correlations. In addition, a principal component analysis revealed four main factors, accounting for 82% of the variance (Visualization, Emotion, Salience, and Exposure). The validity of the Glasgow Norms was established via comparisons of our ratings to 18 different sets of current psycholinguistic norms. The dimension of size was tested with megastudy data, confirming findings from past studies that have explicitly examined this variable. Alternative senses of ambiguous words (i.e., disambiguated forms), when discordant on a given dimension, seemingly led to appropriately distinct ratings. Informal comparisons between the ratings of ambiguous words and of their alternative senses showed different patterns that likely depended on several factors (the number of senses, their relative strengths, and the rating scales themselves). Overall, the Glasgow Norms provide a valuable resource—in particular, for researchers investigating the role of word recognition in language comprehension.

**Electronic supplementary material:**

The online version of this article (10.3758/s13428-018-1099-3) contains supplementary material, which is available to authorized users.

The Glasgow Norms provide a set of normative ratings for 5,553 English words on nine psycholinguistic dimensions. Each word was rated on the dimensions of arousal, valence, dominance, concreteness, imageability, familiarity, age of acquisition, semantic size, and gender association. The aim was to develop a substantial set of standardized, freely available psycholinguistic materials. The norms provide researchers with a considerable collection of materials that are not only reliably evaluated on specific dimensions of interest, but also on other potentially confounding dimensions. Accordingly, the norms allow for the creation and analysis of carefully controlled stimuli, facilitating continued investigations into these lexical dimensions as well as their interactions.

In comparison to previous word norms, the Glasgow Norms offer several significant, novel features. First, a relatively large number of lexical dimensions (nine) was examined. Other norms typically assess only one to three dimensions. Second, the same participant provided ratings across all nine dimensions for any given word. Currently, researchers interested in investigating more than a few lexical dimensions need to access different sets of norms that are tested on different populations of participants. Additionally, as different norms test nonoverlapping word sets, it is often difficult to obtain ratings on all stimuli on all dimensions of interest. Third, two of the dimensions, semantic size and gender association, have not been investigated to date via an extensive set of norms. Finally, many words in the English lexicon are ambiguous, having more than one meaning (e.g., *bank*, having a “money” or “river” sense). The Glasgow Norms include ambiguous words presented in different forms (to different participants)—as isolated words (e.g., *bank*), and as words presented with disambiguating information (e.g., *bank (money)* or *bank (river)*). These key aspects of our approach make the Glasgow Norms a unique and valuable methodological contribution.

There are currently several sets of psycholinguistic norms that report ratings of words on particular psycholinguistic dimensions. Typically, such norms comprise ratings of either 1,000 or so words on a few dimensions, or more than 10,000 words on a single dimension. Table 1 summarizes such norms, limited to those based on more than 500 words. For each set of norms, information is provided about the lexical dimensions examined, the number of words used, and the number of participants tested.

**Table 1:** Inventory of English word norms

Dimension(s)	*N* Items	Participants/Item	Source
AROU, VAL, DOM	1,034	50 on average	Bradley and Lang (1999)
AROU, VAL, DOM	13,915	18–30 for most items	Warriner, Kuperman, and Brysbaert (2013)
CNC	37,058	at least 25	Brysbaert, Warriner, and Kuperman (2014)
IMAG (monosyllabic words)	3,000	31	Cortese and Fugett (2004)
IMAG (disyllabic words)	3,000	35	Schock, Cortese, and Khanna (2012)
CNC, IMAG	925	28 (CNC), 30 (IMAG)	Paivio, Yuille, and Madigan (1968)^a^
CNC, IMAG	1,080	50	Friendly, Franklin, Hoffman, and Rubin (1982)
CNC, IMAG, FAM	2,854	54–65	Toglia and Battig (1978)^b^
CNC, IMAG, FAM, AOA	1,944	35–37	Gilhooly and Logie (1980a)^c^
CNC, IMAG, FAM, AOA (homograph meanings)	905	35–37	Gilhooly and Logie (1980b)
IMAG, FAM, AOA	1,526	20	Stadthagen-Gonzalez and Davis (2006)
IMAG, FAM, AOA	629	21 (IMAG), 14 (FAM), 15 (AOA)	Juhasz, Lai, and Woodcock (2015)^d^
IMAG, FAM	2,311	16 (IMAG), 47–49 (FAM)	Clark and Paivio (2004)^e^
IMAG, AOA	2,694	78 (IMAG), 45 (AOA)	Bird, Franklin, and Howard (2001)
IMAG, AOA	2,204	277	Davies, Izura, Socas, and Dominguez (2016)
AOA (monosyllabic words)	3,000	32	Cortese and Khanna (2008)
AOA (disyllabic words)	3,000	32	Schock, Cortese, Khanna, and Toppi (2012)
AOA	30,124	18–22 for most items	Kuperman, Stadthagen-Gonzalez, and Brysbaert (2012)
AOA (homograph meanings)	3,460	30	Khanna and Cortese (2011)
GEND	600	356	Crawford, Leynes, Mayhorn, and Bink (2004)

For each word norm, the relevant semantic dimension(s), number of words tested, number of participants per item, and citation are specified. Selected word norms comprise those having more than 500 lexical items

*AROU* arousal; *VAL* valence; *DOM* dominance; *CNC* concreteness; *IMAG* imageability (“imagery” in earlier norms); *FAM* familiarity; *AOA* age of acquisition; *GEND* gender association

^a^Paivio et al. also measured meaningfulness. ^b^Toglia and Battig also measured meaningfulness, pleasantness, categorizability, and number of attributes or features. ^c^Gilhooly and Logie (1980a) also measured ambiguity. ^d^Juhasz et al. also measured meaning dominance, semantic transparency, and sensory experience. ^e^Clark and Paivio also measured an additional 13 dimensions, but only on the original set of 925 items from Paivio et al.

It is beyond the scope of the present investigation to catalog norms comprising fewer than 500 lexical items. Nevertheless, over the past several decades, such norms have proved valuable and have been used extensively (e.g., Morrison, Chappell, & Ellis’s, 1997, age of acquisition norms). Oftentimes, however, researchers need to use multiple sets of smaller norms to adequately describe the characteristics of their experimental stimuli (e.g., Scott, O’Donnell, & Sereno, 2012; Sereno, O’Donnell, & Sereno, 2009; Sereno, Scott, Yao, Thaden, & O’Donnell, 2015). Alternatively, researchers have frequently gathered local ratings on their stimuli to ensure the validity of the lexical dimension(s) of interest (e.g., Altarriba, Bauer, & Benvenuto, 1999; Juhasz & Rayner, 2003; Kousta, Vinson, & Vigliocco, 2009; Sereno et al., 2009; Yao et al., 2013, 2018). In other cases, the dimension of interest, although pertinent to the study, is one that is either not widely employed or well-established. For example, researchers have evaluated words on the basis of “context availability” (Schwanenflugel, Harnishfeger, & Stowe, 1988), “danger” and “usefulness” (Wurm, 2007), “offensiveness” and “tabooness” (Janschewitz, 2008), or “body–object interactivity” (Siakaluk, Pexman, Aguilera, Owen, & Sears, 2008).

The Glasgow Norms provide ratings of 5,553 words on nine dimensions: arousal (AROU), valence (VAL), dominance (DOM), concreteness (CNC), imageability (IMAG), familiarity (FAM), age of acquisition (AOA), semantic size (SIZE), and gender association (GEND). The first three dimensions—AROU, VAL, and DOM—are typically used to characterize a word’s emotional impact. AROU is a measure of internal activation (excitement, calmness), VAL is a measure of value or worth (positive, negative), and DOM indicates the degree of control one feels (dominant, controlled). Similar to existing emotion norms (e.g., Bradley & Lang, 1999; Warriner, Kuperman, & Brysbaert, 2013), these are measured on 9-point scales. In the psycholinguistic literature, emotion is generally represented within a two-dimensional framework (e.g., Osgood, Suci, & Tannenbaum, 1957; Russell, 1980), with greater emotionality associated with higher arousal and extreme valence. In behavioral terms, positive and negative emotion words tend to be recognized faster than comparable neutral words (e.g., Scott, O’Donnell, Leuthold, & Sereno, 2009; Scott et al., 2012; Scott, O’Donnell, & Sereno, 2014; Sereno et al., 2015; Yao et al., 2018).

All remaining dimensions of the Glasgow Norms are based on 7-point rating scales, a practice consistent with most existing norms. CNC represents the degree to which something can be experienced by our senses (concrete, abstract). Concrete words are typically recognized faster than abstract words (e.g., Juhasz & Rayner, 2003; Schwanenflugel et al., 1988; Whaley, 1978; Yao et al., 2013, 2018). Kousta, Vigliocco, Vinson, Andrews, and Del Campo (2011), however, proposed that abstract words tend to be more emotionally valenced than concrete words, which gives rise to a residual processing advantage of abstract over concrete words—critically, once opposing effects of context availability and imageability are controlled. IMAG represents the degree of effort involved in generating a mental image of something (imageable, unimageable). In general, imageable words are facilitated in processing as compared to less imageable words (e.g., Balota, Cortese, Sergent-Marshall, Spieler, & Yap, 2004; Cortese & Schock, 2013; Yao et al., 2018). CNC and IMAG, although highly correlated (see, e.g., Paivio, Yuille, & Madigan, 1968), are nevertheless considered to capture distinct semantic aspects of a word (Kousta et al., 2011; Richardson, 1976).

The measures of FAM and AOA are related in different subjective ways to the objective measure of word frequency, in which the relative number of occurrences of individual words within a substantial corpus (more often written than spoken) are calculated (e.g., the British National Corpus, 2007; Davies, 2004). FAM is a measure of a word’s subjective experience (familiar, unfamiliar), and can be partially contrasted with subjective frequency estimates (Balota, Pilotti, & Cortese, 2001), which are considered to be less dependent on other meaning-level variables. Words that are more familiar are recognized faster than those that are less familiar (e.g., Connine, Mullennix, Shernoff, & Yelen, 1990). AOA is a measure of the age at which a word was initially acquired. Although there are alternative ways of measuring AOA (e.g., Juhasz, 2005; Morrison et al., 1997), it is often assessed by adults providing an estimate of when they first learned a word, in spoken or written form, on a 7-point scale (a series of 2-year periods from 0–12 years and a final 13+ period). Zevin and Seidenberg (2002, 2004) suggested that our developmental experience with words may be better captured by measures of their cumulative frequency (summed lifetime usage) and frequency trajectory (how usage changes over time). More recently, however, Brysbaert (2017) demonstrated that the best predictor of objective AOA is rated AOA. Behaviorally, words acquired earlier in life are recognized faster than those acquired later (e.g., Cortese & Khanna, 2007; Johnston & Barry, 2006; Juhasz & Rayner, 2006; Sereno & O’Donnell, 2009).

The final dimensions of SIZE and GEND have only been the subject of more recent psycholinguistic investigations (e.g., Sereno & O’Donnell, 2009; Sereno et al., 2009; Yao et al., 2013). SIZE is a measure of magnitude (big, small) expressed in either concrete or abstract terms. That is, words can refer to objects or concepts that are considered bigger (e.g., *castle*, *wealth*) or smaller (e.g., *pocket*, *unique*). It has been demonstrated that words referring to bigger things are recognized faster than those referring to smaller ones (e.g., Sereno et al., 2009; Yao et al., 2013). GEND is a measure of the degree to which words are considered to be associated with male or female behavior (masculine, feminine). Recent norms have specifically examined gender perception of role nouns across languages (Garnham, Doehren, & Gygax, 2015; Misersky et al., 2014). Although reading studies have investigated gender role stereotypes (e.g., *electrician*, *secretary*) and their gendered mis/matching pronouns (e.g., Duffy & Keir, 2004; Kreiner, Sturt, & Garrod, 2008), there has been little if any research into gender associations to a much broader spectrum of content words. Sereno and O’Donnell (2009) investigated words rated as either male- or female-oriented (e.g., *frog*, *cigar*, *guitar* or *duck*, *flute*, *heaven*, respectively) in a lexical decision task (AOA was also manipulated). They found that whereas female participants demonstrated an advantage to same-gendered words (e.g., responses were faster to *tights* than *pliers*), male participants showed no such comparable bias (i.e., *pliers* was no faster than *tights*).

The present corpus of 5,553 words includes a range of content words (nouns, verbs, adjectives, adverbs) as well as 379 semantically ambiguous words (homographs) whose alternative meanings were additionally rated. To our knowledge, only a few existing norms have explicitly included ambiguous words. Clark and Paivio (2004), in their extension of the Paivio et al. (1968; *N* = 925) norms, included number of meanings as an additional measure, but did not collect ratings on the alternative meanings, themselves. Bird, Franklin, and Howard (2001) did acquire IMAG and AOA ratings on a subset of their items (*N* = 110) of noun–verb homographs (disambiguated by preceding the ambiguous word with *a* or *to*, respectively). Gilhooly and Logie (1980a) had participants rate whether or not their words had multiple meanings, resulting in a set of ambiguous words (*N* = 649) that were then further rated for the relative dominance of alternative senses. Gilhooly and Logie (1980b) collected ratings on a set of 387 ambiguous words having a total of 905 separate meanings on the scales of CNC, IMAG, FAM, and AOA. Khanna and Cortese (2011) collected AOA ratings of 1,208 ambiguous words having a total of 3,460 senses. Although most ambiguous words are “biased,” having a strongly dominant meaning and one or more subordinate meanings, some are “balanced,” having two more salient meanings, with other possible subordinate senses (Sereno, O’Donnell, & Rayner, 2006). Ratings of homographs from previous norming studies that have *not* explicitly disambiguated their disparate senses probably reflect participants’ interpretation of the dominant meaning, although this is not a certainty. The ambiguous words identified in the Glasgow Norms were presented alone (e.g., *ball*), or in disambiguated form (e.g., *ball (sphere)* or *ball (dance)*), critically, to different participants.

The Glasgow Norms were collected by presenting our corpus of 5,553 words to participants in lists of either 101 or 150 words. For each list, participants rated words separately on all nine dimensions described above. The relations among dimensions are explored and comparisons to other norms are provided.

## Method

### Participants

A profile detailing the number, age, and gender of participants is presented in Table 2. A total of 829 individuals (“unique participants”) took part in the rating studies, with some completing more than one word list. When participants were tallied on the basis of completing a single list (“all participants”), regardless of whether they had completed other lists, the total came to 1,368. Overall, their ages ranged from 16 to 73 years, and there were slightly more than twice the number of females than males who took part. The participants were native English speakers from the University of Glasgow community and were recruited opportunistically via an experiment advertisement link on the home page of the Psychology department at the University of Glasgow. They were either paid at a rate of £6/h or given course credit for their participation. The study conformed to British Psychological Society ethical guidelines and protocols.

**Table 2:** Age and gender profile of participants

	Unique participants			All participants	
	*N* (%)	Age (*SD*)	# Lists (*SD*)	*N* (%)	Age
Female	599 (72)	21.5 (7.6)	1.6 (1.3)	960 (70)	22.6
Male	230 (28)	22.3 (6.9)	1.8 (1.6)	409 (30)	23.5
All	829 (100)	21.7 (7.4)	1.7 (1.4)	1,368 (100)	22.8

The number of participants, age, and average number of lists completed are provided by grouping and gender. “Unique participants” comprise individuals, some of whom provided ratings for more than one list of words; “all participants” represent the total number of participants responding to all lists, and does not take into account whether any given participant took part in more than one list. The majority of participants, 69%, completed a single list of words. The remaining percentages of individual participants completing more than one list are as follows: 17% did two lists, 3% did three lists, 8% did four lists, and 3% did eight lists

### Materials

A corpus of 5,553 words was assembled from an initial set of 808 words and a subsequent, larger set of 4,800 words (with 55 words included in both lists). The data acquired from these two sets were merged into a single corpus for subsequent analyses. Words ranged in length from two to 16 letters, with an average length of 6.10 letters (*SD* = 1.99).

The corpus included 379 ambiguous words. Each was presented in isolation or with disambiguating information following the word in parentheses (e.g., *solution*, or *solution (answer)* and *solution (chemical)*, respectively). The average number of disambiguated forms presented was 2.30 (*SD* = .58). The number of words having two, three, four, and five alternative meanings was 289, 69, 19, and 2, respectively. Thus, a total of 871 items in the corpus were presented with disambiguation.

### Procedure

The experiment was run online via an in-house experimental platform (http://experiments.psy.gla.ac.uk). Each participant rated a list of either 101 (eight possible lists of the 808 word set) or 150 words (32 lists of the 4,800 word set). Lists of 101 or 150 words were generated by taking every 8th or 32nd item from alphabetized versions of either the 808 or 4,800 sets, respectively. This way, each list was representative of the set in terms of its distribution of word-initial letters and no list contained more than one instance of any given ambiguous word in any of its forms.

The general instructions for the experiment, the specific instructions for each of the nine different rating tasks, and the rating scale labels are presented in the supplementary materials to this article in Tables S1, S2, and S3, respectively. The same participant provided ratings across all nine dimensions for any given word. Participants rated all words of a list on one scale, then all words on the next scale, and so on. The order of words within each scale was randomized as was the order of scales across participants. The approximate time to complete the experiment was 40 or 60 min for 101- or 150-item lists, respectively.

## Results

Data were eliminated from further analyses if the response time (RT) to any word on any scale was less than 600 ms or if participants reported not knowing a word. For RT, examination of the trial-by-trial data revealed the presence of infrequent episodes of rapid responding by some participants, typically repeating a given rating value. In such cases, the RTs tended to be less than 400 ms. In two recent, large-scale lexical decision experiments performed locally, average RTs to words were just under 600 ms (Sereno et al., 2015, used 240 words with 144 participants; Yao et al., 2018, used 270 words with 127 participants). A conservative lower cutoff of 600 ms was therefore implemented in the present study. No upper cutoff was imposed. While participants were encouraged to rate each word according to their initial interpretation of its meaning, there was no emphasis on speed of response. That is, participants were not instructed, for example, to respond to each item as quickly as possible. An identical procedure of not implementing an upper cutoff has been employed by several sets of norms (e.g., Clark & Paivio, 2004; Cortese & Khanna, 2008; Khanna & Cortese, 2011; Schock, Cortese, & Khanna, 2012). Of the total number of responses recorded (*N* = 1,732,607), the RT distribution was as follows: 3.07% were shorter than 600 ms, 77.68% were 600–3,000 ms (with 36.03% 1,500–2,000 ms), 12.98% were 3,000–5,000 ms, and 6.27% were longer than 5,000 ms. In terms of word knowledge, for all scales except for FAM, if participants did not know the meaning of a word, they could select the “unfamiliar word” button instead of rating it (see the instructions in the Table S2). This option accounted for 0.33% of all responses. On average, 33.29 responses were provided per word (*SD* = 3.76). A detailed profile of the numbers of responses across all nine rating scales is presented in Table 3.

**Table 3:** Profile of the numbers of responses across dimensions

*N*		AROU	VAL	DOM	CNC	IMAG	FAM	AOA	SIZE	GEND
5,553	*M*	33.31	33.54	33.24	33.34	33.30	32.36	33.94	33.30	33.25
	*SD*	3.72	3.73	3.73	3.80	3.74	3.60	3.69	3.79	3.85
	range	13–70	15–71	14–69	11–70	14–70	22–67	17–70	13–70	15–69
55	*M*	65.73	65.96	65.31	65.33	64.84	61.18	66.75	65.96	64.64
	*SD*	1.57	1.55	2.01	2.16	2.43	3.20	1.51	1.96	2.44
	range	61–70	63–71	61–69	60–70	59–70	53–67	64–70	60–70	59–69
5,498	*M*	32.99	33.22	32.92	33.02	32.99	32.07	33.61	32.98	32.94
	*SD*	1.83	1.85	1.90	2.05	2.01	2.15	1.68	1.92	2.22
	range	13–36	15–36	14–36	11–36	14–36	22–35	17–36	13–36	15–36

Profile of the number of responses to the overall corpus (*N* = 5,553), the subset of words (*N* = 55) repeated across the 808- and 4,800-word lists, and the majority of words (*N* = 5,498) presented in only one of the two lists

*AROU* arousal; *VAL* valence; *DOM* dominance; *CNC* concreteness; *IMAG* imageability; *FAM* familiarity; *AOA* age of acquisition; *SIZE* semantic size; *GEND* gender association

The descriptive statistics for the nine rated dimensions are presented in Table 4. The Glasgow Norms are available as part of the supplementary materials to this article and are provided in .csv format. The Glasgow Norms present an alphabetized list of 5,553 words. The columns, from left to right, are as follows: word, length (which excludes possible disambiguating information), and, for each of the nine dimensions, the mean rating (*M*), standard deviation (*SD*), and number of ratings (*N*) for each word. Ratings for the 55 words that were included in both lists were highly correlated for all scales, ranging from *r* = .88 for DOM to *r* = .97 for VAL.

**Table 4:** Descriptive statistics of the nine dimensions of the Glasgow Norms

Dimension	Scale range	*M*	*SD*
AROU	1–9	4.63	1.10
VAL	1–9	5.10	1.55
DOM	1–9	5.07	0.91
CNC	1–7	4.64	1.42
IMAG	1–7	4.79	1.35
FAM	1–7	5.26	0.93
AOA	1–7	4.13	1.24
SIZE	1–7	4.09	1.02
GEND	1–7	4.12	0.91

Scale ranges and mean (*M*) and standard deviation (*SD*) values for the nine psycholinguistic dimensions of the Glasgow Norms

*AROU* arousal; *VAL* valence; *DOM* dominance; *CNC* concreteness; *IMAG* imageability; *FAM* familiarity; *AOA* age of acquisition; *SIZE* semantic size; *GEND* gender association

### Relations between the nine dimensions of the Glasgow Norms

To provide an initial overview of the relations between all nine of the Glasgow Norms scales, we performed Spearman correlations, and these are presented in Table 5. Since Spearman correlations are rank-based, this method takes into account both linear and nonlinear relations between the dimensions. We used the Bonferroni method to correct *p* values for multiple tests and applied a significance threshold of *p* = .01. Due to the large number of items (*N* = 5,553), almost all correlations were significant. However, considering only large effects (i.e., with *r*s > .5; Cohen, 1988), the following correlations were particularly strong: CNC × IMAG (*r* = .91; the more concrete a word is, the easier it is to imagine); VAL × DOM (*r* = .68; the more positive a word is, the more it provokes feelings of dominance); FAM × AOA (*r* = – .67; the more familiar a word is, the earlier that word was learned); and SIZE × AROU (*r* = .51; the bigger the object or concept is to which a word refers, the more arousing the word is).

**Table 5:** Correlations between dimensions of the Glasgow Norms

	AROU	VAL	DOM	CNC	IMAG	FAM	AOA	SIZE	GEND
AROU	**–**								
VAL	.35	**–**							
DOM	.34	**.68**	**–**						
CNC	– .25	(.05)	(.05)	**–**					
IMAG	– .10	.10	.08	**.91**	**–**				
FAM	.18	.30	.23	.10	.22	**–**			
AOA	(.00)	– .19	– .14	– .38	– .49	**– .67**	**–**		
SIZE	**.51**	.12	.09	– .41	– .33	(.05)	.22	**–**	
GEND	– .11	– .42	– .09	.15	.07	– .21	.15	.15	**–**

Spearman coefficients for all combinations of scales. All correlations are significant (*p* < .01; Bonferroni corrected), except those listed in parentheses. Tests printed in bold are those considered large, with *r*s >|±.50| (Cohen, 1988)

*AROU* arousal; *VAL* valence; *DOM* dominance; *CNC* concreteness; *IMAG* imageability; *FAM* familiarity; *AOA* age of acquisition; *SIZE* semantic size; *GEND* gender association

For a more detailed analysis of relations between scales, we fit linear and quadratic models to the data, using the MATLAB function fitlm (The MathWorks, Inc.). To account for outliers, the fits were computed using a robust least-squares method (bisquare weighting function). Reported *R*^2^s were adjusted for the number of coefficients. The results for all combinations of dimensions using linear and quadratic fits are included in the supplementary materials to this article as Tables S4 and S5, respectively.

**Fig. 1:**
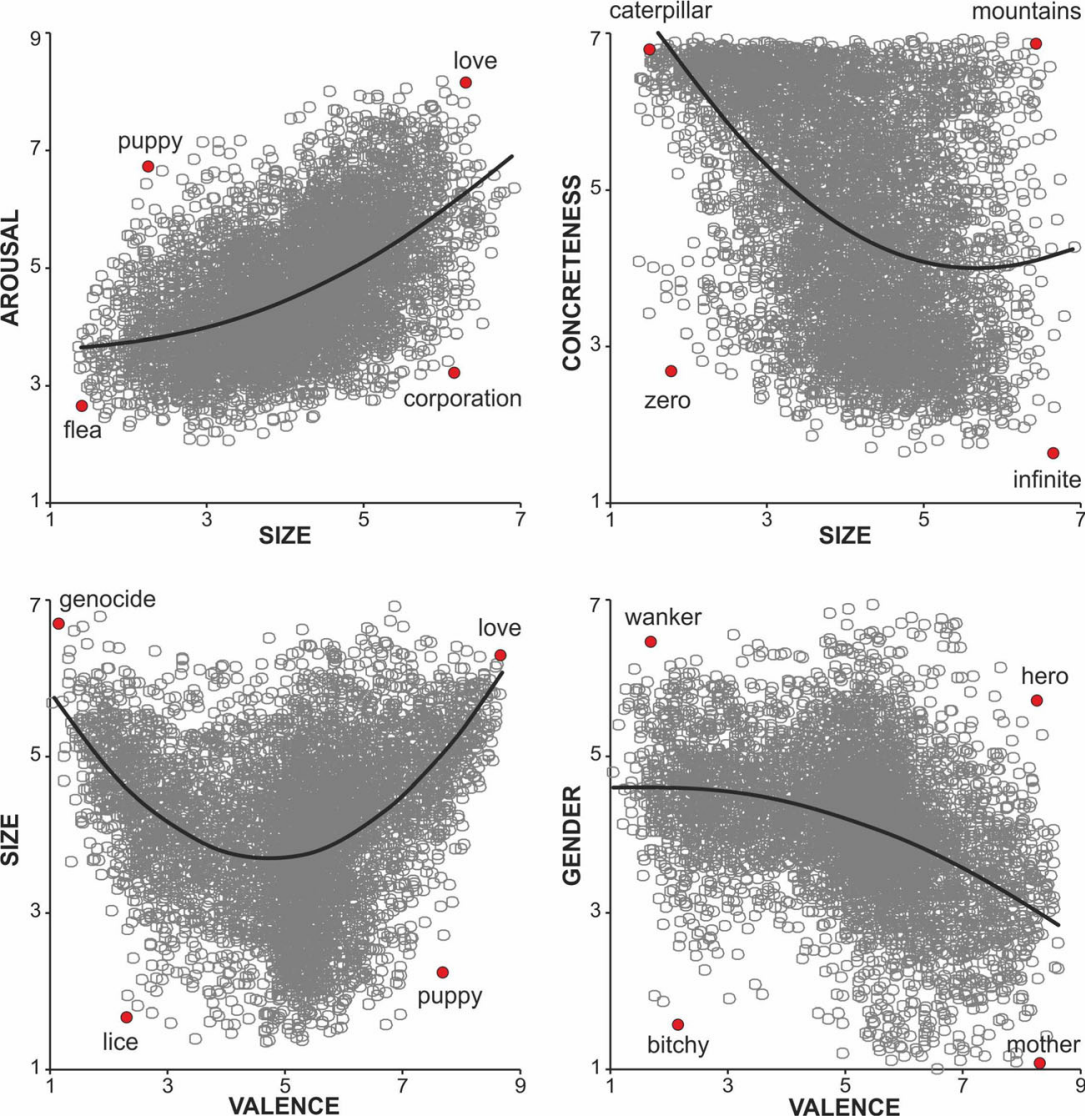
Quadratic fits with semantic size or gender association that explain more than 18% of variance. *R*^2^ values, *F* values, and significance for the linear and quadratic fits for all combinations of dimensions can be found in Tables S4 and S5 of the supplementary materials

We will highlight the effects of SIZE and GEND, as these two dimensions are relatively new and less well understood. Figure 1 shows the quadratic fits for all combinations with either SIZE or GEND that account for more than 18% of variance (see Table S5): SIZE × AROU (*R*^2^ = .27), SIZE × CNC (*R*^2^ = .19), VAL × SIZE (*R*^2^ = .19), and VAL × GEND (*R*^2^ = .18). For three of these, the linear fits accounted for comparable (but numerically slightly less) amounts of variance (see Table S5) and are straightforward to interpret: SIZE × AROU (the semantically bigger a word is, the more arousing it is); SIZE × CNC (the semantically bigger a word is, the less concrete it is); and VAL × GEND (the more positive a word is, the more feminine it is). In contrast, VAL × SIZE was explained better by a quadratic (*R*^2^ = .19) than by a linear (*R*^2^ < .01) fit (the more extremely valenced—negative *or* positive—a word is, the semantically bigger it is).

### Factor analysis of dimensions

To summarize and interpret the correlation results and explore the relative alignment of the newer scales of SIZE and GEND, we performed a factor analysis. The data for all nine scales were submitted to a principal component analysis (PCA) with an oblique rotation (direct oblimin; Harman, 1976; Jennrich & Sampson, 1966). Note that using an orthogonal rotation (e.g., varimax, quartimax, or equamax) yielded comparable results. Factors with eigenvalues greater than 1 were included in the factor solution (Kaiser, 1960).

The factor analysis is presented in Table 6 and yielded a solution with four factors. The first factor, Visualization, accounted for 30% of the variance in the data and included the scales CNC and IMAG. The second factor, Emotion, accounted for an additional 26% of the variance and included VAL and DOM. The third and fourth factors each accounted for 13% of the variance: a Salience factor, including SIZE, GEND, and AROU, and an Exposure factor, including FAM and AOA. Together, the four factors explained 82% of the common variance. The communality for each scale was above .6, indicating that the amount of variance accounted for by the retained factors was sufficient. In other words, the scales’ variance was useful in delineating the extracted factors.

**Table 6:** Factor loadings for all dimensions of the Glasgow Norms

	Factor 1:	Factor 2:	Factor 3:	Factor 4:
	Visualization	Emotion	Salience	Exposure
CNC	**.938**	.098	– .083	– .053
IMAG	**.888**	.116	– .013	– .220
VAL	.025	**.919**	– .163	– .016
DOM	.170	**.914**	.101	.103
SIZE	– .344	.086	**.753**	– .074
GEND	.446	– .283	**.683**	.238
AROU	– .242	.443	**.521**	– .211
FAM	– .026	– .036	.081	**– .935**
AOA	– .286	.068	.087	**.843**
%Var	29.77	25.87	13.18	12.97
%CumVar	29.77	55.64	68.82	81.79

Reported are loadings of an oblique rotation matrix (direct oblimin) on four factors. Loadings >|±.50| are highlighted in bold. Explained common variance (%Var) is given for individual factors, as well as the cumulative variance (%CumVar)

*CNC* concreteness; *IMAG* imageability; *VAL* valence; *DOM* dominance; *SIZE* semantic size; *GEND* gender association; *AROU* arousal; *FAM* familiarity; *AOA* age of acquisition

It is noteworthy that most of the scales loaded relatively high (i.e., above an absolute value of .5) on one factor. However, AROU (loading highest on the factor Salience) also loaded on the factor Emotion, and GEND (also loading highest on the factor Salience) additionally loaded on the factor Visualization, indicating that these variables cannot be explained in terms of a single factor.

### Correlations with other psycholinguistic norms

To confirm the validity of our ratings, we correlated the dimensions of the Glasgow Norms with 18 of the 20 different sets of English norms listed in Table 1. The norms that were excluded were those that were not easily accessible. Between one and ten norms were available for all dimensions except SIZE. Because linear relations between norms were expected, we performed Pearson correlations for all shared words. These correlations are presented in Table 7. All correlations were highly significant (*p*s < .0001, Bonferroni corrected), and the vast majority showed a Pearson coefficient greater than .5, indicating a large effect (Cohen, 1988) and, therefore, sufficient validity. We do not have an explanation for why two of the 36 correlations reported in Table 7, although significant, had coefficients less than .5. The highest and most consistent correlations were achieved by VAL, CNC, AOA, and GEND (with nearly all *r*s > .9).

**Table 7:** Correlations between the Glasgow Norms and other English word norms

Norms	*N*_source_	*N*_overlap_	AROU	VAL	DOM	CNC	IMAG	FAM	AOA	GEND
1	1,034	951	**.66**	**.95**	**.82**					
2	13,915	4,073	**.62**	**.93**	**.69**					
3	37,058	4,445				**.93**				
4	3,000	1,363					**.88**			
5	3,000	1,308					**.89**			
6	925	789				**.93**	**.92**			
7	1,944	902				**.93**	**.88**	**.82**	**.92**	
8	905	136				**.81**	**.84**	**.72**	**.86**	
9	1,526	1,370					**.92**	**.81**	**.94**	
10	629	61					**.94**	**.64**	**.90**	
11	2,311	1,390					.42	**.82**		
12	2,694	994					**.80**		**.86**	
13	2,204	722					**.91**		**.95**	
14	3,000	1,363							**.91**	
15	3,000	1,308							**.90**	
16	30,124	4,283							**.89**	
17	3,460	525							.20	
18	600	336								**.96**

Pearson coefficients for 18 sets of norms reporting scales corresponding to the Glasgow Norms (note that no norms were available for semantic size). For each of the norms, the number of total items (*N*_source_) and the number of identical items within the Glasgow Norms (*N*_overlap_) that were used for the correlations are indicated. Norms 8 and 17 examine different senses of ambiguous words. All correlations were highly significant (*p*s < .0001, Bonferroni corrected). Correlations with a large effect (*r* > .5, see Cohen, 1988) are printed in bold. References for the 18 norms are as follows: 1 = Bradley and Lang (1999); 2 = Warriner et al. (2013); 3 = Brysbaert et al. (2014); 4 = Cortese and Fugett (2004); 5 = Schock, Cortese, and Khanna (2012); 6 = Paivio et al. (1968); 7 = Gilhooly and Logie (1980a); 8 = Gilhooly and Logie (1980b); 9 = Stadthagen-Gonzalez and Davis (2006); 10 = Juhasz et al. (2015); 11 = Clark and Paivio (2004); 12 = Bird et al. (2001); 13 = Davies et al. (2016); 14 = Cortese and Khanna (2008); 15 = Schock, Cortese, et al. (2012); 16 = Kuperman et al. (2012); 17 = Khanna and Cortese (2011); and 18 = Crawford et al. (2004)

*AROU* arousal; *VAL* valence; *DOM* dominance; *CNC* concreteness; *IMAG* imageability; *FAM* familiarity; *AOA* age of acquisition; *SIZE* semantic size; *GEND* gender association

### SIZE and GEND

One unique strength of our norms is the inclusion of the SIZE and GEND variables, which allows us to test these effects on a much larger set of words than had previously been possible. To assess the effects of SIZE, we attempted to replicate the semantic size effect reported in Sereno et al. (2009) and Yao et al. (2013). We combined our ratings and the lexical decision RT data from the English Lexicon Project (ELP; Balota et al., 2007). To avoid multiple entries of the same word, we removed items corresponding to the alternative meanings of homographs. A total of 4,568 words were entered into the analysis. We examined the effects of SIZE on RTs, with all the other variables as covariates (word frequency, word length, CNC, IMAG, AROU, VAL, FAM, AOA, DOM, and GEND). To address collinearity between the covariates (e.g., CNC × IMAG, AROU × VAL), we reduced the dimensions of covariates via a PCA using a varimax rotation. We extracted seven principal components, accounting for 93.8% of the variance, and their factor loadings are shown in Table 8. We fit a linear model of RT with SIZE and the extracted principal components as predictors, and the results are shown in Table 9. After we had controlled for a wide range of lexical and semantic variables, SIZE negatively predicted word recognition times—that is, semantically bigger words were recognized significantly faster than semantically smaller words, replicating the findings of Sereno et al. (2009) and Yao et al. (2013).

**Table 8:** SIZE Effects: Factor Loadings for the Extracted Principal Components

	PC1	PC2	PC3	PC4	PC5	PC6	PC7
Frequency			**0.98**				0.16
Length	–0.10	0.10				**0.97**	–0.13
CNC	**0.95**	–0.22					
IMAG	**0.96**						0.19
AROU		**0.81**		0.45			
|VAL|	–0.21	**0.88**		–0.22			
FAM		0.11	0.10	0.10			**0.94**
AOA	–0.35		–0.12			0.31	**–0.79**
DOM				**0.96**			0.11
GEND					**0.99**		–0.10

*Note*: Factor loadings ≥ |±.10| are shown and factor loadings > |±.50| are highlighted in bold. Frequency was measured in written occurrences per million as per the British National Corpus (2007; Davies, 2004) and Length in number of letters

*CNC* concreteness; *IMAG* imageability; *AROU* arousal; |VAL| absolute valence (i.e., the 1-9 scale was collapsed around its midpoint so that higher values would reflect more extreme valence, regardless of whether they were rated as positive or negative); *FAM* familiarity; *AOA* age of acquisition (negative loading); *DOM* dominance; *GEND* gender association; *SIZE* semantic size

**Table 9:** SIZE Effects: Corresponding Multiple Linear Regression Results

Predictors	*b*	*SE*	*t*	*p*
SIZE	–10.51	1.21	–8.68	<.001
PC1 (CNC, IMAG)	–5.99	0.96	–6.21	<.001
PC2 (AROU, |VAL|)	4.35	1.08	4.04	<.001
PC3 (Frequency)	–7.28	0.89	–8.17	<.001
PC4 (DOM)	–3.99	0.91	–4.39	<.001
PC5 (GEND)	3.65	0.93	3.91	<.001
PC6 (Length)	40.44	0.94	43.01	<.001
PC7 (FAM, -AOA)	–45.44	0.90	–50.3	<.001

*Note*: Frequency was measured in written occurrences per million as per the British National Corpus (2007; Davies, 2004) and Length in number of letters

*CNC* concreteness; *IMAG* imageability; *AROU* arousal; |*VAL*| absolute valence (i.e., the 1-9 scale was collapsed around its midpoint so that higher values would reflect more extreme valence, regardless of whether they were rated as positive or negative); *FAM* familiarity; *AOA* age of acquisition (negative loading); *DOM* dominance; *GEND* gender association; *SIZE* semantic size

The effects of word GEND, however, are more difficult to test. RTs in megastudies are aggregated across participant gender. Moreover, the relative proportion of male versus female participants in megastudies is typically not specified. Sereno and O’Donnell (2009) examined the effects of word GEND and AOA on lexical decision times across male and female participants. All participants demonstrated AOA effects. However, females took longer to respond to male-oriented words, particularly late-AOA ones, whereas males, in contrast, demonstrated no effect of word GEND. Confirming this pattern of results with megastudy data (in combination with our norms) would entail finding a GEND × AOA interaction across participant gender. Without access to data that separately present responses from male and female participants, we were unable to directly test the effects of word GEND.

### Ambiguous words

**Fig. 2:**
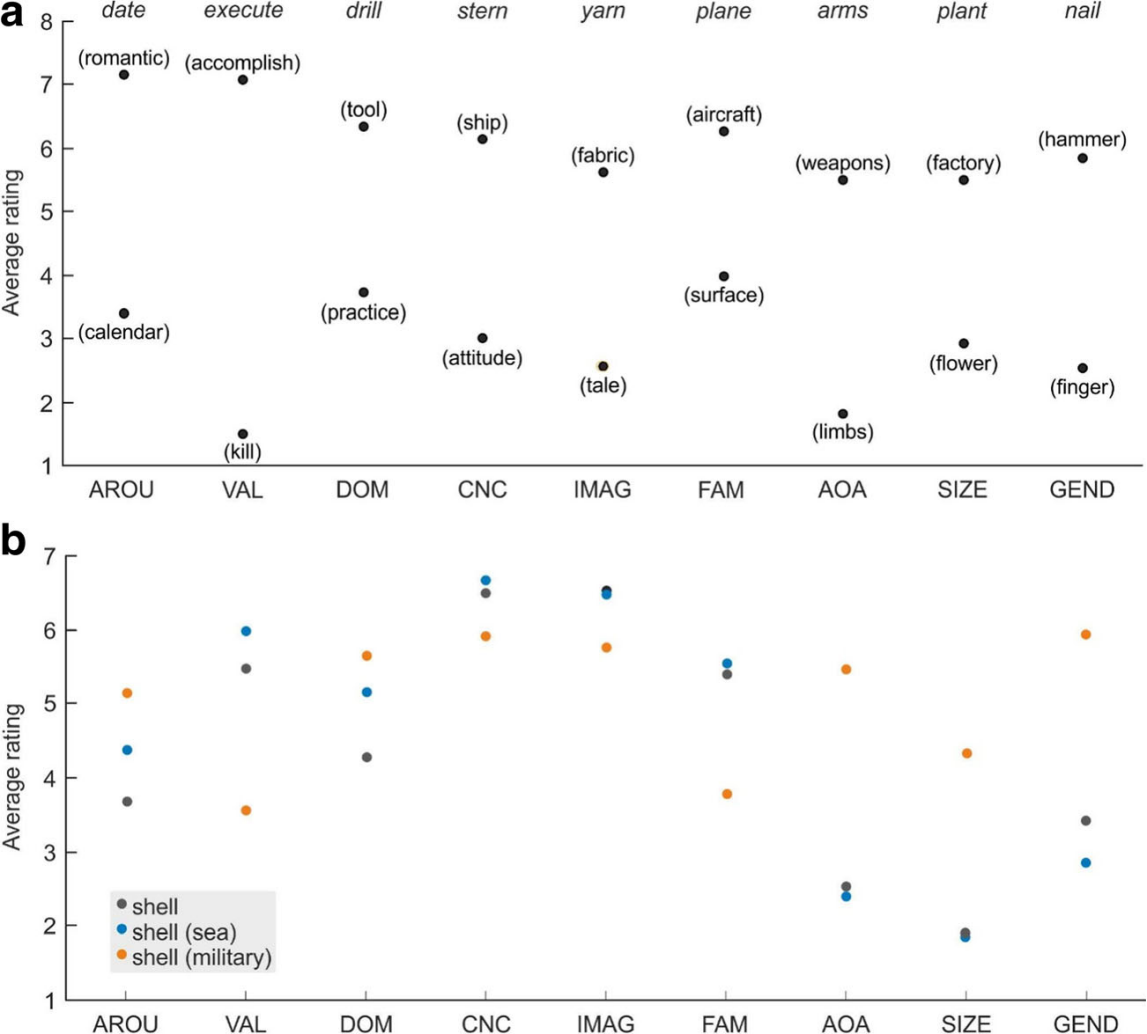
(**a**) Ratings of alternative senses of ambiguous words are indicated across dimensions. For each dimension of the Glasgow Norms, an example ambiguous word is listed across the top. The ratings corresponding to the alternative senses (defined in parentheses) are indicated beneath each ambiguous word. (**b**) Ratings of the ambiguous word *shell* and its alternative senses (“sea” and “military”) are indicated across all dimensions of the Glasgow Norms. *AROU* arousal; *VAL* valence; *DOM* dominance; *CNC* concreteness; *IMAG* imageability; *FAM* familiarity; *AOA* age of acquisition; *SIZE* semantic size; *GEND* gender association

We did not perform any formal analyses on the ratings of ambiguous words in the corpus, whether they occurred in isolation (e.g., *pen*) or in disambiguated form (e.g., *pen (ink)*, *pen (cage)*). Informal examination of the ratings, however, indicated certain patterns. First, when disambiguating information was provided, the alternative senses of ambiguous words received distinct ratings where they were relevant to the dimension in question. Figure 2A illustrates the ratings that alternative senses of several ambiguous words received across the nine dimensions—in particular, where the alternative senses were expected to lead to disparate judgments. The second aspect of ambiguous word ratings concerned the relationship between a word’s ambiguous and disambiguated forms. Although ambiguous words typically have a dominant and one or more subordinate senses (Sereno et al., 2006), the relative strengths of these alternative senses can vary substantially, not only across items, but across individuals. It is also possible that the dimensions themselves may have served as “contexts” for ambiguous words presented in isolation (i.e., given the scale AROU, a participant may have rated *plot* in its “story” sense, but later, given the scale CNC, rated *plot* in its “land” sense, because these were the most accessible meanings). Figure 2B shows the ratings across all dimensions of the ambiguous word *shell* and two of its alternative senses (“sea” and “military”). Although ratings for *shell* tended to be closer to its dominant “sea” sense than its subordinate “military” sense, they did not always overlap as might be expected. Moreover, we observed several different patterns of ratings across ambiguous items. Oftentimes it did not appear that ambiguous words were rated according to only one of their senses. Without an independent measure of the dominance relationships among the alternative senses of ambiguous words in our corpus, however, we are at present unable to characterize these data. Anecdotally, factors such as number of meanings, their relative strengths, and the rating scale itself seem to play distinct roles.

Two sets of prior norms contained a fairly large number of ambiguous words with clearly defined alternative meanings that were rated on a subset of dimensions (Gilhooly & Logie, 1980b; Khanna & Cortese, 2011). We correlated the shared dimensions of the disambiguated words from these samples with the disambiguated items from our norms whose meanings matched. These correlations were significant and are included in Table 7 (Norms 8 and 17).

## Discussion

The Glasgow Norms examined nine semantic dimensions of words in a corpus of 5,553 words, with an average of 33 participants contributing ratings to each word on each scale. Seven of the dimensions (AROU, VAL, DOM, CNC, IMAG, FAM, and AOA) are well-established and have been investigated extensively, whereas the other two dimensions (SIZE and GEND) are relatively novel and have not been examined in a comprehensive way. In comparison to past norms, the Glasgow Norms provide an internally consistent set of ratings, not only across a sizeable corpus, but also across a considerable set of psycholinguistic dimensions. Moreover, the Glasgow Norms provide ratings for a significant number of ambiguous words (*N* = 379), presented in isolation (e.g., *figure*) as well as in disambiguated forms (e.g., *figure (body shape)*, *figure (graph)*, *figure (number)*, and *figure (reckon)*).

Analyses comprised evaluating the relations among the dimensions in the Glasgow Norms and comparing its results to those of other norms. Correlations between the nine dimensions of the Glasgow Norms were generally significant (see Table 5) due to the large number of items. Particularly strong relationships (with *r*s > .5; Cohen, 1988) included the following: CNC × IMAG (concrete words are easier to imagine); VAL × DOM (positive words provoke greater feelings of dominance); FAM × AOA (familiar words are acquired earlier); and SIZE × AROU (words referring to bigger things are more arousing). The first three relationships are established in the literature (e.g., Bradley & Lang, 1999; Friendly et al., 1982; Gilhooly & Logie, 1980a; Paivio et al., 1968; Stadthagen-Gonzalez & Davis, 2006; Toglia & Battig, 1978; Warriner et al., 2013). The latter is a novel finding, although it has already obtained behavioral support (Yao et al., 2013). In further analyses, we fit linear and quadratic models for all combinations of the dimensions (see Tables S4 and S5 in the supplementary materials). We focused on effects related to the relatively new dimensions of SIZE and GEND (see Fig. 1). For SIZE, words referring to bigger things were more arousing, more extremely (positively or negatively) valenced, and more abstract. For GEND, feminine words were more positive. It should be noted that although all participants were native English speakers, we did not record whether they were fluent in any other languages. Knowledge of a grammatically gendered language may have potentially impacted GEND ratings of English words in some participants (Boroditsky, Schmidt, & Phillips, 2003).

Factor analysis of all dimensions of the Glasgow Norms yielded a four-factor solution accounting for 82% of the variance (see Table 6). The factors and their associated high-loading dimensions were as follows: Visualization (CNC, IMAG), Emotion (VAL, DOM), Salience (SIZE, GEND, AROU), and Exposure (FAM, AOA). Notably, both AROU “and GEND also loaded moderately on Emotion and Visualization, respectively.” The lack of a one-to-one mapping between factors and dimensions highlights both the complexity of these semantic relationships as well as the need to recognize their potential influence in the design and analysis of psycholinguistic research.

The validity of the Glasgow Norms was assessed by a series of correlations with 18 different sets of English norms (see Table 7). All dimensions of the Glasgow Norms were tested with the exception of SIZE (to our knowledge, we are the first to obtain extensive ratings for this dimension). For any given dimension, between one and seven comparisons were made to previous sets of norms. The correlations with the prior norms were highly significant across the eight dimensions of AROU, VAL, DOM, CNC, IMAG, FAM, AOA, and GEND.

The newer dimensions of SIZE and GEND were tested against megastudy data from the ELP (Balota et al., 2007). Analyses revealed that both SIZE and GEND effects were obtained, confirming the findings from prior studies that have specifically examined these factors.

Finally, the Glasgow Norms included a set of 379 ambiguous words—presented alone or with disambiguating information. This is the first time an appreciable number of ambiguous words as well as their alternative senses have been normed across an extensive number of lexical dimensions. Informal examination of the data demonstrated that alternative senses of ambiguous words having contrasting meanings were rated appropriately (see Fig. 2A). Ambiguous words presented in isolation were sometimes rated according to their highly dominant sense across the different dimensions (see Fig. 2B). In general, however, the rating patterns we observed for ambiguous words and their disambiguated senses varied. We believe that these different configurations most likely depend on several factors, including the number of alternative senses, the dominance relationship among these senses, as well as the rating scales, themselves.

In conclusion, the Glasgow Norms represent a valuable resource, providing a substantial set of words normed across a large number of psycholinguistic dimensions. Key features of the norms include the evaluation of the relations between dimensions, the validation of established dimensions, the assessment of novel dimensions, and the examination of ambiguous words and their meanings. Use of the Glasgow Norms will allow both the manipulation and control of lexical variables, in particular, in studies that investigate word recognition processes, whether in the experimental context of word-based tasks or during the course of fluent reading. Establishing the semantic contingencies and interactions of these variables will inform models of language processing.

## Electronic supplementary material

ESM 1 (PDF 167 kb)

ESM 2 (CSV 795 kb)

ESM 3 (PDF 64.7 kb)
