# Supplementary material for: The Glasgow Norms: Ratings of 5,500 words on nine scales
Source: Behav Res Methods. 2018 Sep 11;51(3):1258–70. doi: 10.3758/s13428-018-1099-3 (PMC6538586; doi:10.3758/s13428-018-1099-3)
Supplement: Supplementary file 1 — (PDF 167 kb) [file 13428_2018_1099_MOESM1_ESM.pdf]

Instructions for Rating Tasks

We have included all of the instructions for the norming task. The general instructions are presented in **Table S1**. The specific instructions for each of the nine separate rating tasks, including how each variable was defined, are presented in **Table S2**. Finally, the specifications of each rating scale in terms of the number of scale points and scale labels are presented in **Table S3**.

Table S1
General Instructions

In this experiment, you will be rating a set of 101/150 words on 9 different scales – familiarity, concreteness, arousal, valence, dominance, imagaeability, size, gender, and age of acquisition. These different scales assess different aspects of word meanings.

You will rate the entire word set on one scale, then rate them all again on the next scale, and so on. You will be given instructions about what each scale represents before you begin each scale.

Sometimes words have more than one meaning – for example, the word "nail" has one meaning related to fingers and one related to hammers In such cases, we will display the word in one of its meanings – "nail (finger)" or "nail" (hammer)" – or just by itself as "nail". Please rate these words according to your first impression.

It is also possible that you may be presented with a word that you don't know. If this happens, there is a button "Unfamiliar word" located below the rating scale that you can click on to proceed to the next trial.

Finally, it may sometimes be difficult to rate a word on a given scale. For example, the word "desk" might be difficult to categorise as either being a masculine or feminine thing. Likewise, the word "amusing" might be difficult to categorise as something that is big or small. When you are faced with such difficult decisions, please respond as best as you can without thinking too deeply – go with your intuitions. Note: Click on the red information button in the upper right corner at any time to refer to the rating instructions again.

Note: One version of the ratings included 101 words; the other included 150.

Table S2
The Nine Rating Scales

Arousal

Arousal is a measure of excitement versus calmness. A word is AROUSING if it makes you feel stimulated, excited, frenzied, jittery, or wide-awake. A word is UNAROUSING if it makes you feel relaxed, calm, sluggish, dull, or sleepy.

Please indicate how arousing you think each word is on a scale of VERY UNAROUSING to VERY AROUSING, with the midpoint representing moderate arousal.

Valence

Valence is a measure of value or worth. A word is POSITIVE if it represents something considered good, whereas a word is NEGATIVE if it represents something considered bad.

Please indicate the valence of each word on a scale of VERY NEGATIVE to VERY POSITIVE, with the midpoint representing NEUTRAL.

Dominance

Dominance is a measure of the degree of control you feel. A word can make you feel DOMINANT, influential, in control, important, or autonomous. Conversely, a word can make you feel CONTROLLED, influenced, cared-for, submissive, or guided.

Please indicate how each word makes you feel on a scale of YOU ARE VERY CONTROLLED to YOU ARE VERY DOMINANT, with the midpoint being neither controlled nor dominant.

Concreteness

Concreteness is a measure of how concrete or abstract something is. A word is CONCRETE if it represents something that exists in a definite physical form in the real world. In contrast, a word is ABSTRACT if it represents more of a concept or idea.

Please indicate how concrete you think each word is on a scale of VERY ABSTRACT to VERY CONCRETE, with the midpoint being neither especially abstract nor concrete.

Imageability

Imageability is a measure of how easy or difficult something is to imagine. A word is IMAGEABLE if it represents something that is very easy to imagine or picture. In contrast, a word is UNIMAGEABLE if it represents something that is very difficult to imagine or picture.

Please indicate how imageable you think each word is on a scale of VERY UNIMAGEABLE to VERY IMAGEABLE, with the midpoint being moderately imageable.

Familiarity

Familiarity is a measure of how familiar something is. A word is very FAMILIAR if you see/hear it often and it is easily recognisable. In contrast, a word is very UNFAMILIAR if you rarely see/hear it and it is relatively unrecognisable.

Please indicate how familiar you think each word is on a scale of VERY UNFAMILIAR to VERY FAMILIAR, with the midpoint representing moderate familiarity.

Age of Acquisition

A word's age of acquisition is the age at which that word was initially learned. Please estimate when in your life you think you first acquired or learned each word. That is, try to remember how old you were when you learned each word either in its spoken or written form (whichever came first).

The scale is defined as a series of consecutive 2-year periods from the ages of 0 to 12 years, and a final period encompassing 13 years and older.

Semantic Size

Size is a measure of something's dimensions, magnitude, or extent. A word represents something BIG if it refers to things or concepts that are large. A word represents something SMALL if it refers to things or concepts that are little.

Please indicate the semantic size of each word on a scale of VERY SMALL to VERY BIG, with the midpoint being neither small nor big.

Gender Association

A word's gender is how strongly its meaning is associated with male or female behaviour. A word can be considered MASCULINE if it is linked to male behaviour. Alternatively, a word can be considered FEMININE if it is linked to female behaviour.

Please indicate the gender associated with each word on a scale of VERY FEMININE to VERY MASCULINE, with the midpoint being neuter (neither feminine nor masculine).

Note: For each scale, the definition of each variable provided in the instructions are indicated. All scale definitions ended with the closing statement, "In your ratings, please note the following: there are no correct or incorrect answers – each person will have differing opinions; trust your intuitions – do not spend a long time thinking about each word as your initial reaction is often the most accurate; and try to use the whole range of the scale.

Table S3
Specifications of Rating Scales

Rating Scale	Number of Scale Points	Scale Labels (left to right)
Arousal	9	Very Unarousing, Very Arousing
Valence	9	Very Negative, Neutral, Very Positive
Dominance	9	You are very Controlled, You are very Dominant
Concreteness	7	Very Abstract, Very Concrete
Imageability	7	Very Unimageable, Very Imageable
Age of Acquisition	7	0-2, 3-4, 5-6, 7-8, 9-10, 11-12, 13+
Semantic Size	7	Very Small, Very Big
Gender Association	7	Very Feminine, Very Masculine

Note: For all scales, endpoints were labelled as indicated. Additional labels were as follows: Valence included a midpoint label; Age of Acquisition included labels for each scale point.
