# Supplementary material for: The Glasgow Norms: Ratings of 5,500 words on nine scales
Source: Behav Res Methods. 2018 Sep 11;51(3):1258–70. doi: 10.3758/s13428-018-1099-3 (PMC6538586; doi:10.3758/s13428-018-1099-3)
Supplement: Supplementary file 3 — (PDF 64.7 kb) [file 13428_2018_1099_MOESM3_ESM.pdf]

Table S4
Linear Fits between All Dimensions of the Glasgow Norms

	**AROU**		**VAL**		**DOM**		**CNC**		**IMAG**		**FAM**		**AOA**		**SIZE**		**GEND**	
	R^2	F	R^2	F	R^2	F	R^2	F	R^2	F	R^2	F	R^2	F	R^2	F	R^2	F
**AROU**			0.19	1365.98	0.15	1008.04	0.07	412.40	0.01	58.85	0.04	214.54	(0.00)	(0.05)	0.27	2010.73	0.02	92.60
**VAL**	0.10	602.43			0.55	6840.56	0.00	18.80	0.01	44.33	0.07	392.73	0.03	185.50	0.01	28.13	0.17	1103.56
**DOM**	0.12	734.25	0.57	7413.74			0.00	7.94	0.00	25.27	0.04	242.32	0.02	88.18	0.01	42.05	0.01	32.87
**CNC**	0.07	391.68	0.01	30.96	0.00	19.95			0.85	31109.71	0.01	67.94	0.14	874.54	0.18	1200.09	0.03	166.34
**IMAG**	0.01	57.87	0.01	58.18	0.01	41.23	0.85	31207.00			0.04	248.52	0.24	1701.88	0.11	711.38	0.01	56.07
**FAM**	0.03	190.48	0.07	406.42	0.04	247.96	0.01	49.00	0.04	253.92			0.45	4519.46	(0.00)	(10.83)	0.04	213.96
**AOA**	(0.00)	(7.56)	0.04	203.21	0.02	108.58	0.13	855.51	0.23	1667.76	0.45	4465.91			0.05	283.53	0.02	124.70
**SIZE**	0.26	1920.04	0.02	89.35	0.01	54.59	0.17	1158.89	0.11	718.23	0.01	31.91	0.05	302.70			0.03	144.88
**GEND**	0.01	86.68	0.15	1019.34	0.01	39.52	0.02	89.00	0.00	16.64	0.04	215.19	0.02	103.57	0.02	125.23		

Note: Reported are R^2s and F-values (with *df* 1 and 5,552). All R^2s are significant (p<.01; Bonferroni corrected) except those listed in parentheses. AROU=arousal; VAL=valence; DOM=dominance; CNC=concreteness; IMAG=imageability; FAM=familiarity; AOA=age of acquisition; SIZE=semantic size; and GEND=gender association.

Table S5
Quadratic Fits between All Dimensions of the Glasgow Norms

	**AROU**		**VAL**		**DOM**		**CNC**		**IMAG**		**FAM**		**AOA**		**SIZE**		**GEND**	
	R^2	F	R^2	F	R^2	F	R^2	F	R^2	F	R^2	F	R^2	F	R^2	F	R^2	F
**AROU**			0.23	815.15	0.18	593.55	0.07	206.39	0.01	34.75	0.04	110.56	(0.00)	(1.56)	0.27	1011.57	0.02	68.05
**VAL**	0.40	1826.61			0.55	3426.36	0.13	431.31	0.05	150.24	0.09	262.30	0.03	94.65	0.19	671.23	0.18	626.67
**DOM**	0.23	818.17	0.57	3726.15			0.10	302.56	0.05	160.46	0.05	151.32	0.02	53.41	0.11	325.59	0.01	16.63
**CNC**	0.07	202.51	0.01	32.24	0.01	25.10			0.85	15549.17	0.03	71.13	0.15	475.52	0.19	630.99	0.04	110.44
**IMAG**	0.02	47.40	0.02	47.65	0.01	30.68	0.85	15927.66			0.05	154.48	0.25	906.19	0.14	448.92	0.01	34.44
**FAM**	0.03	96.68	0.08	251.35	0.05	153.52	0.01	29.66	0.05	130.26			0.48	2551.04	(0.00)	(5.43)	0.04	128.83
**AOA**	(0.00)	(4.06)	0.04	101.60	0.02	55.27	0.13	428.17	0.23	834.30	0.46	2363.87			0.05	153.18	0.03	74.57
**SIZE**	0.28	1052.73	0.04	111.85	0.02	43.28	0.19	649.81	0.15	489.33	0.02	53.35	0.07	213.81			0.03	74.09
**GEND**	0.06	174.78	0.16	510.62	0.01	40.01	0.05	155.08	0.07	198.76	0.04	107.64	0.02	59.97	0.02	66.54		

Note: Reported are R^2s and F-values (with *df* 2 and 5,552). All R^2s are significant (p<.01; Bonferroni corrected) except those listed in parentheses. AROU=arousal; VAL=valence; DOM=dominance; CNC=concreteness; IMAG=imageability; FAM=familiarity; AOA=age of acquisition; SIZE=semantic size; and GEND=gender association.
